# A non-invasive approach to explore the discriminatory potential of the urinary volatilome of invasive ductal carcinoma of the breast†

**DOI:** 10.1039/c8ra02083c

**Published:** 2018-07-12

**Authors:** Khushman Taunk, Ravindra Taware, Tushar H. More, Priscilla Porto-Figueira, Jorge A. M. Pereira, Rajkishore Mohapatra, Dharmesh Soneji, José S. Câmara, H. A. Nagarajaram, Srikanth Rapole

**Affiliations:** Proteomics Lab, National Centre for Cell Science Ganeshkhind Pune 411007 India rsrikanth@nccs.res.in +91-20-2569-2259 +91-20-2570-8075; CQM – Centro de Química da Madeira, Universidade da Madeira, Campus Universitário da Penteada Funchal 9000-390 Portugal; Laboratory of Computational Biology, Centre for DNA Fingerprinting & Diagnostics (CDFD) Nampally Hyderabad 500001 India; Malignant Disease Treatment Centre, Military Hospital (Cardio Thoracic Centre), Armed Forces Medical College Pune 411040 India; Faculdade de Ciências Exatas e da Engenharia, Universidade da Madeira, Campus Universitário da Penteada Funchal 9000-390 Portugal; Department of Biotechnology & Bioinformatics, School of Life Sciences, University of Hyderabad Hyderabad 500 046 India

## Abstract

Worldwide, breast invasive ductal carcinoma (IDC) accounts for the majority of the reported cases of this form of cancer. IDC effective management, as for any form of cancer, would greatly benefit from early diagnosis. This, however, due to various socio-economic reasons, is very far for the reality in developing countries like India, where cancer diagnosis is often carried out at late stages when disease management is troublesome. With the present work, we aim to evaluate a simple analytical methodology to identify a set of volatile organic compounds (VOCs) in urine samples, as a biosignature for IDC. Using solid-phase microextraction followed by gas chromatography/mass spectrometry, a panel of 14 urinary VOCs was found to discriminate IDC (*n* = 65) from a healthy control (HC) group (*n* = 70) through multivariate statistical treatments. Furthermore, metabolic pathway analysis revealed various dysregulated pathways involved in IDC patients hinting that their detailed investigations could lead to novel mechanistic insights into the disease pathophysiology. In addition, we validated the expression pattern of five of these VOCs namely 2-ethyl-1-hexanol, isolongifolenone, furan, dodecanoic acid, 2-methoxy-phenol in another external cohort of 59 urinary samples (IDC = 32 and HC = 27) and found their expression pattern to be consistent with the primary sample set. To our knowledge, this is the first study exploring breast IDC volatome alterations in Indian patients.

## Abstract 2

A non-invasive urinary volatilomics approach for exploring the IDC type breast cancer.

## Graphical abstract

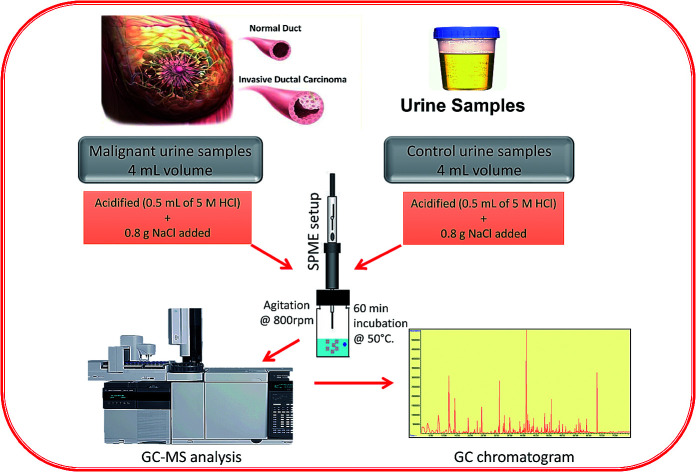

## 1. Introduction

The global burden of Breast Cancer (BC) related mortality among women follows an exponential pattern.^1,2^ Breast cancer exists as a major public health problem affecting women worldwide. In 2013, around 14.9 million BC cases and 8.2 million deaths have been reported.^1,3^ In developing countries like India, BC mortality rates follow the global trend and late diagnosis and advanced disease presentation are also hallmarks for this scenario.^4^ Being a heterogeneous disease, BC has been grouped into different types according to the histological and molecular characteristics.^5,6^ About 60–80% of the reported cases relate to the most prevalent type of BC otherwise known as invasive ductal carcinoma (IDC), which is the abnormal tumorous growth in the breast ductal epithelium which is capable of invading the surrounding tissues, through a phenomenon known as metastasis.^7,8^

The key to successfully treat one of the deadliest non-communicable diseases, cancer and in this case breast IDC, lies in the fact that it should be diagnosed correctly and at a very early stage during the onset of cancer. However, the current oncological diagnostic procedures are invasive, expensive and need expert medical staffs to evaluate the severity of the disease. Nevertheless, the widespread usage of diagnostic techniques such as mammography and other imaging techniques has undoubtedly helped to reduce the women mortality related with BC. One of the major demerits, however, is the high frequency of false negative results incurred across different age groups and high failure rates towards detection of tumor amongst young women who have dense breast tissue, which decreases mammographic sensitivity.^9–11^

Developing countries, like India, thrive to have a higher frequency of IDC mortalities majorly due to lack of regular health check-ups amongst the women, expensive diagnostic tests and scarcity of cancer screening methods for large populations. Thus, new operational strategies able to be easily and massively implemented in the real clinical settings towards BC screening are urgently needed. Many molecular entities such as genes, proteins and metabolites have been proposed as biomarkers for BC. However, besides their limited performance, the identification of these biomolecules is usually carried out from body fluids or tissues of the patients, often using invasive and expensive procedures. Platforms based on ‘omics’ technologies have been explored extensively towards many diseases including cancer^12–15^ even at subtype levels.^16^ In cancer research, metabolomics is emerging at a rapid pace with the advancement in mass spectrometry and other analytical technologies. Metabolomics is the study of a complete set of metabolites expressed in a living system, which are the final products of the complete biological processes. Therefore, the identification of alterations in metabolite levels through various metabolomics approaches holds great potential to delineate and detect heterogeneous varied oncological diseases at early stage.^17^ The different physiological processes pertaining to tumor growth and metastasis needs various mechanisms of tissue remodeling favored by numerous metabolic readjustments, thereby indicating a deep close-knit network between cancer and metabolites.^18–20^ Metabolomic alterations have also been reported as an initial cause of cancer occurrence^20^ and various researchers have proposed differential expression of small molecule metabolites in a variety of cancer through metabolomics approaches.^21–27^

As a subset of metabolomics, many researchers have explored the potential of volatile organic compounds (VOCs) in a variety of biological samples related to numerous diseases.^28–34^ It could be highly desirable to the patients and health care systems if the samples needed for cancer screening tends to be obtained non-invasively. Furthermore, VOCs could be a potential indicator of the disease state as they are easily available in non-invasive samples like urine, saliva, *etc.* The panel of VOC based biosignature could be effectively implemented towards early diagnosis of IDC, which could be helpful to detect the tumor development at the onset of cancer progression. Previous research studies have demonstrated that tissues produced unique VOCs and revealed that the VOC concentrations change during pathologic states, including infection, neoplasia, or metabolic disease.^35–38^ Currently, the biomedical researchers are also trying to identify this kind of biomarkers in non-invasive patient samples, which may ease the physical pain of the patients. Many studies have used the VOC profile present in such non-invasive body fluids for differentiating patients from healthy controls (HC). The most suitable body fluid for analysis of VOCs would be urine due to the fact that the compounds from the total metabolism of the body are concentrated by the kidney before getting excreted *via* urine,^39^ thereby making urine as a rich source of metabolites. Câmara and co-workers reported BC-associated VOCs in urine and demonstrated the advantage of non-invasive VOCs signature.^40^ Recently, Silva *et al.* explored the VOC alterations in BC using different cell lines.^34^ However, there are no reports on VOC based biosignature for IDC type of BC, which is a high prevalence subtype worldwide. In this study, we aim to explore and identify the urinary VOCs alterations in IDC towards potential non-invasive biosignature, which can be helpful to clinicians to diagnose the IDC at an early stage. To our knowledge, this is the first report to identify urinary VOCs associated with IDC in the Indian context.

## 2. Materials and methods

### 2.1 Subject selection, sample collection and storage

IDC samples were collected from the Malignant Disease Treatment Centre (MDTC), Unit of the Military Hospital-Cardio Thoracic Centre (MH-CTC), Armed Forces Medical College (AFMC), Pune, India. The institutional ethics committee of AFMC and National Centre for Cell Science (NCCS) approved this study. All the participants in this study were informed about the investigation and informed consent approval was obtained from the patients prior to sample collection following the Declaration of Helsinki guidelines (DoH, 2008). The inclusion criteria for this study included the recruitment of only such women patients who had minimum 18 years of age, were devoid of hypertension and diabetes, were histologically confirmed for IDC and those who did not undergo any anticancer therapeutic interventions. The age and gender-matched healthy women devoid of hypertension, diabetes and not on any medication regime for the last three months were recruited as healthy controls (HC). The controls were confirmed by physical examination at the clinic for not having any breast lumps or lesions. Samples from healthy controls were obtained through the health check-up camp organized by the MDTC, MH-CTC, AFMC, Pune. Post-fasting condition first-morning urine samples (in 50 mL sterile tubes) from 65 IDC patients (average age 54 ± 8) and 70 HC individuals (average age 50 ± 10) were collected and utilized for this study. The clinical and demographic information of the subjects is summarized in Table S1.† The samples were labeled, centrifuged at 5000 × *g* for 10 min at 4 °C, filtered through 0.45 μm syringe filters and stored at −80 °C until further analysis within two hours of collection.

### 2.2 Sample preparation

Head Space Solid Phase Micro Extraction (HS-SPME) technique was employed to extract the VOCs present in the urine samples of the subjects as demonstrated elsewhere.^40–42^ In brief, 4 mL of urine samples was transferred to 8 mL headspace sampling glass vials (Thermo Fisher, USA) having a small magnetic stirring bar. The urine samples were subjected to acidification by adding 500 μL HCl (5 M) and further 0.8 g NaCl (both Merck, Germany) was also added to the acidified urine samples to enhance the extraction of VOCs in the headspace of vials. The efficiency of the extraction of urinary VOMs enhances if the metabolites are protonated, which will occur in acidic conditions.^40,41,43^ The vials with processed urine were crimped using aluminum caps having PTFE/silicone septa to make this an isolated system. These airtight sealed vials were subjected to incubation at 50 ± 1 °C with continuously stirred at 800 rpm on an in-house made water bath kept on a heated magnetic stirrer (Tarsons, India). To extract the VOCs accumulated in the headspace region of the sample vials, carboxen/polydimethylsiloxane (CAR/PDMS) SPME fibre, 75 μm (Supelco, USA) was immediately exposed for 60 min. The incubated SPME fibre was carefully retracted into its safety needle and exposed back into the 250 °C heated inlet port of the GC-MS system for 6 min for thermal desorption of the VOCs onto the GC-MS column.

### 2.3 Analysis and data processing of VOCs

An Agilent 7890B gas chromatograph (Palo Alto, USA) coupled to an Agilent 5977A quadrupole inert mass selective detector was used to chromatographically separate, detect and identify the urinary VOCs extracted from the IDC and healthy subjects. The complex mixture of urinary VOCs was separated using a BP-20 (SGE, Germany) fused silica capillary column (60 m × 0.25 mm × 0.25 μm) installed in the oven of the gas chromatograph (GC). The GC was operated under the following temperature gradient, for a total GC run time of 87 min, starting from 45 °C held for 5 min, then gradually ramped at 2 °C min^−1^ up to 150 °C with a 10 min hold time and then again increased at 15 °C min^−1^ up to 220 °C and held for 15 min. Ultra high purity helium gas (99.999%, Prama Instruments, India) was employed as mobile phase/carrier gas for the GC with a flow rate of 1 mL min^−1^. The manual injections of the VOC enriched SPME assembly was carried out in splitless mode with 250 °C as the inlet port temperature. All the samples were acquired in duplicates. 250 °C, 150 °C and 230 °C respectively were the operating temperatures of the transfer line, quadrupole and electron impact ionization source. The acquisition of data was performed in full scan mode in the mass range of 30 to 300 *m*/*z* and 70 eV was applied for the electron impact to record the mass spectra. The Agilent ChemStation data analysis software (Palo Alto, USA) coupled with the NIST11 mass spectral library was employed for the identification of the metabolites. Metabolite identification hits from the library search were considered as a confirmatory hit if the match score was ≥ 80%. Further, chromatogram integration to generate peak areas was carried out using ChemStation data analysis software. The C8–C20 *n*-alkanes series were also analysed under the same experimental conditions to obtain the reference retention indices and confirm the identity of the volatiles identified by comparison with the Kovats indices available in the literature for similar experimental conditions. The VOCs that showed missing values >80% across all the samples were removed from further analysis.

### 2.4 Statistical analysis

The statistical data analysis was performed in order to identify the most significant and differentially regulated IDC urinary VOCs from the pool of identified VOCs. MetaboAnalyst 3.0 ^44^ and SIMCA 14.1 packages were used for this purpose. As the urinary VOC data matrix was not primarily under a normal distribution, data normalization was carried out using Metaboanalyst 3.0. The data was quantile normalized, cube root transformed, and range scaled to transform it to follow a normal Gaussian distribution. Quantile normalization methodology thrives to achieve the similar distribution of metabolic feature abundances across all the sample sets.^45^ The data was transformed to the cube root of the data values. Range scaling scales the data to the centered mean and then divide the same by the range of each variable thereby, scaling the features by the variation of biological samples.^46^ Range scaling facilitates the equal consideration of all the variations among the metabolites present in the dataset.^47^ Univariate and multivariate statistical analyses were further performed on the normalized data matrix. Statistical significance was tested using student's *t*-test and Mann Whitney *U* test (both *p* ≤ 0.05) in conjunction with fold change (FC) (≥1.5/≤0.67) analysis and executed on the data to build a panel of statistically significant differentially regulated urinary VOCs between IDC patients and HC individuals. Multivariate statistical treatments performed by SIMCA 14.1 software, comprised unsupervised and supervised model building in order to segregate IDC from HC group. The unsupervised mathematical modeling comprised of principal component analysis (PCA) wherein, the principal components calculated from the data matrix were used primarily to detect intrinsic data clustering about the orthogonal principle components as well as to get a preliminary idea of the outliers present in the study population. Further, upon excluding the outliers observed in the PCA, the data matrix was subjected to different model building tests like partial least squares-discriminant analysis (PLS-DA) and orthogonal partial least squares-discriminant analysis (OPLS-DA) to check for the clustering pattern between IDC and HC group.^48^ The VIP scores generated from the OPLS-DA model were indicative of the VOCs that were most influential (VIP score ≥ 1.0) in segregating IDC from HC group. The *R*^2^ and *Q*^2^ values from the OPLS-DA model were employed to evaluate the quality and reliability of mathematical model generated, in which the *R*^2^ value indicates the goodness of fit and the *Q*^2^ value represents predictability of the model.^49^ To visualize the clustering of the two groups under this study, hierarchical cluster analysis (HCA) was carried out.

### 2.5 Metabolic pathway annotation

Pathway analysis, a module within the MetaboAnalyst 3.0 package, which is a combination of Metabolite set enrichment analysis (MSEA) and pathway topology analysis, was undertaken to identify the altered biochemical pathways in IDC.^50,51^ This module performs the enrichment of the metabolite sets for *Homo sapiens* based upon several libraries, which contain approximately 6300 metabolite sets. In order to compare various pathways, the centrality measures calculated node importance values are normalized further by the sum of the importance of the pathway. Hence, the measure of importance of each metabolite node is actually the percentage with respect to the total pathway importance, and the pathway impact is the cumulative percentage from the matched metabolite nodes.^44,50,51^ The list of VOCs, identified as statistically significant and differentially regulated, was uploaded in the enrichment analysis module to identify the enriched biochemical pathways.

### 2.6 Validation of VOCs with an external cohort

The existence of some of the statistically significant and differentially regulated VOCs which had VIP score ≥ 1.0, were further validated in another external cohort of samples comprising of 32 IDC and 27 HC subjects. High purity analytical standards (Sigma Aldrich) were purchased and were subjected to SPME extraction under same conditions as mentioned in Section 2.2 and further analyzed by GC-MS to determine the retention time (RT) and fragmentation pattern. Based on these criteria, a selected ion monitoring (SIM) method was developed wherein; only specific ions could be monitored in the samples. The presence of five such statistically important VOCs identified in the discovery phase was validated in an independent cohort of IDC urine samples through the SIM mode acquisition of the SPME extracted samples.

## 3. Results

### 3.1 Alterations in the urinary VOCs of IDC identified by GC-MS

**Fig. 1:**
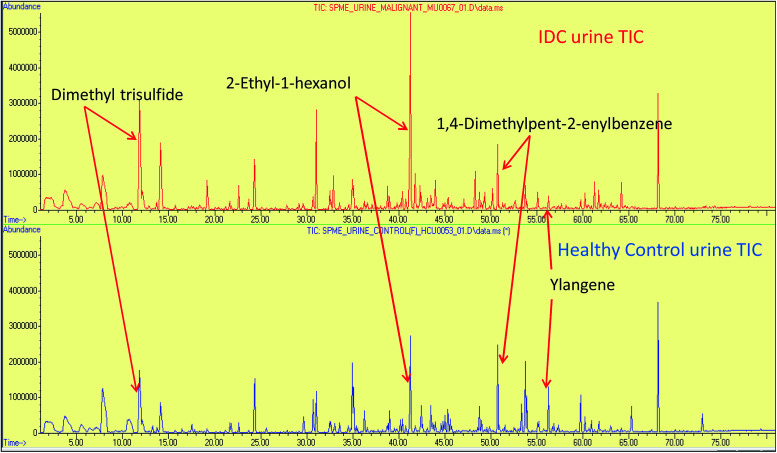
The representative full scan chromatograms of IDC and HC urine samples showing differential regulation of some of the statistically significant VOCs.

The urine samples of 65 IDC patients and 70 HC individuals were analyzed *via* the HS-SPME extraction coupled to GC-MS analysis to establish their urinary VOC profiles. The identity of the VOCs present in the urine samples was confirmed by comparing the mass spectral matches against the NIST11 mass spectral library. The VOCs that had a match score of >80% and had an occurrence frequency of >80% were specifically selected to build the data matrix for further statistical treatment. Abiding the aforementioned criteria, 94 VOCs were qualified from a total of 110 NIST library identified compound hits. The identified VOCs belongs to a variety of different chemical families, mainly benzene derivatives, alcohols, alkanes, sulphur and nitrogen containing compounds, ketones, phenol derivatives, furan derivatives, terpenes, organic acids, aldehydes*.* The list of all the VOCs identified in IDC subjects and healthy controls is enlisted in ESI Table S3.† Representative chromatograms showing differential regulation of some of the VOCs are depicted in Fig. 1.

### 3.2 Statistical treatment of the VOCs identified in IDC and HC group

**Fig. 2:**
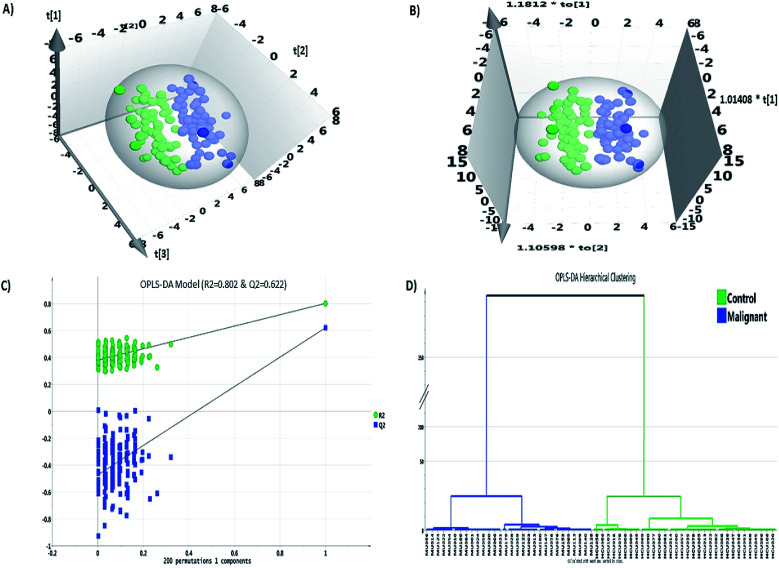
Multivariate statistical analysis for IDC and healthy controls VOC dataset through SIMCA. (A) PLS-DA score plot discriminating the IDC and healthy controls, (B) OPLS-DA score plot discriminating the IDC and healthy controls, (C) permutation test analysis of the OPLS-DA model depicting a valid model; *Y*-axis intercepts: *R*^2^ = 0.802, *Q*^2^ = 0.622, (D) hierarchical clustering analysis between the IDC and healthy controls separates the two groups.

Statistical analysis was carried out using Microsoft Excel 2016, MetaboAnalyst 3.0 and SIMCA 14.1 software packages. The data matrix comprised of 94 VOC features grouped across 135 samples resulting in 12 093 data points including missing values (MV). The MV imputation feature in the MetaboAnalyst 3.0 was used to fill in the 4.7% MVs found in the data matrix. MV imputation of the data was carried out by Bayesian principal component analysis (bPCA) which is one of the most suitable strategies for metabolomics data sets.^52^ Post MV imputation, the data matrix comprising 94 VOCs, was subjected to data normalization as described in Section 2.4, the representative figure of which is depicted in ESI Fig. S1.† The combination of univariate and multivariate statistical tests abiding the cut-off value criteria (FC ≥ 1.5/≤0.67; *p*-value ≤ 0.05 and VIP score ≥ 1.0) revealed a panel of 14 VOCs (Table 1) that were statistically significant, differentially regulated and could discriminate IDC from HC group. The fold change analysis revealed 11 VOCs to be up-regulated whereas three VOCs showed a pattern of down-regulation. 2-Ethyl-1-hexanol and isolongifolenone showed higher than two-fold up-regulation. Multivariate modeling through PLS-DA and OPLS-DA model revealed a good separation cluster between IDC and HC group (Fig. 2a and b). The permutation test for OPLS-DA model, based on 200 random permutations was calculated, which depicted the validity accuracy of the model to discriminate IDC from HC group. The permutation plot distinctly depicts that the *R*^2^ (0.802) and *Q*^2^ (0.622) values of the original OPLS-DA model are well above the permutated models, which indicates that the model is not over-fitted and has the higher predictive ability (Fig. 2c). The HCA plot shows distinct clustering of malignant and healthy controls with no overlapping among the samples in each group (Fig. 2d).

**Table 1:** The panel of 14 statistically significant and differentially regulated VOCs identified through combination of univariate and multivariate statistical tests abiding the cut-off value criteria (FC ≥ 1.5/≤0.67; *p*-value ≤ 0.05 and VIP score ≥ 1.0) that discriminated IDC from HC group. Sorted according to the FC values in decreasing order. RT: retention time, KI_expt_: experimental Kovat's index, KI_theo_: theoretical Kovat's index

S. No.	Differentially expressed VOCs (common names)	Differentially expressed VOCs (NIST11 names)	VIP score	FC value	log2 FC value	Student's *t*-test *p*-value	Mann-Whitney *U* test *p*-value	RT	KI_expt_	KI_theo_
1	2-Ethyl-1-hexanol	1-Hexanol, 2-ethyl-	1.19	6.41	2.68	1.50 × 10^−3^	1.17 × 10^−2^	39.33	1423	1453
2	Isolongifolenone	2,4*a*-Methanonaphthalen-7(4*aH*)-one, 1,2,3,4,5,6-hexahydro-1,1,5,5-tetramethyl-, 2*s-cis*-	1.75	2.41	1.27	2.76 × 10^−3^	4.56 × 10^−3^	75.125	2090	—
3	*m*-Cresol	Phenol, 3-methyl-	1.11	1.89	0.92	9.27 × 10^−3^	3.86 × 10^−3^	72.497	2009	2060
4	Acetic acid	Acetic acid	1.30	1.86	0.90	2.08 × 10^−3^	3.58 × 10^−5^	37.672	1396	1400
5	2,2,7,7-Tetramethyltricyclo[6.2.1.0(1,6)]undec-4-en-3-one	2,2,7,7-Tetramethyltricyclo[6.2.1.0(1,6)]undec-4-en-3-one	1.49	1.86	0.90	2.12 × 10^−2^	9.50 × 10^−3^	73.229	2031	—
6	*p*-Cresol	Phenol, 4-methyl-	1.02	1.79	0.84	1.17 × 10^−2^	3.80 × 10^−3^	72.314	2003	2031
7	Phenol	Phenol	1.12	1.79	0.83	1.63 × 10^−3^	2.83 × 10^−3^	70.382	1941	1946
8	Guaiacol	Phenol, 2-methoxy-	1.38	1.76	0.82	1.90 × 10^−3^	3.57 × 10^−3^	60.916	1785	1815
9	Furan	Furan	1.78	1.61	0.68	4.10 × 10^−3^	6.12 × 10^−6^	5.541	889	831
10	Dimethyl trisulfide	Dimethyl trisulfide	1.05	1.60	0.68	1.26 × 10^−2^	5.78 × 10^−3^	31.423	1301	1332
11	Dodecanoic acid	Dodecanoic acid	1.08	1.59	0.67	1.31 × 10^−3^	1.52 × 10^−3^	81.067	2263	2446
12	Ylangene	Ylangene	1.37	0.60	−0.73	3.33 × 10^−2^	4.42 × 10^−2^	46.71	1543	1518
13	1,4-Dimethylpent-2-enylbenzene	1,4-Dimethylpent-2-enylbenzene	1.12	0.59	−0.76	5.62 × 10^−3^	3.91 × 10^−2^	34.296	1347	—
14	1-4-Hydroxy-3,5-di-*tert*-butylphenyl-2-methyl-3-morpholinopropan-1-one	1-4-Hydroxy-3,5-di-*tert*-butylphenyl-2-methyl-3-morpholinopropan-1-one	1.61	0.47	−1.08	1.02 × 10^−2^	2.57 × 10^−2^	78.998	2204	—

### 3.3 Validation of the differentially expressed VOCs in an external cohort using GC-MS in SIM mode

**Fig. 3:**
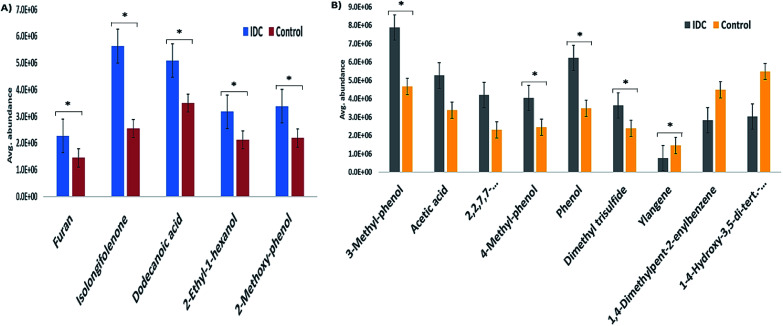
The bar graph representing the expression profile of significantly altered VOCs as seen in the external cohort of subjects. (A) The five verified VOCs with SIM mode GC-MS analysis and (B) the semi-quantitative chromatographic areas graph for the nine VOCs. The bars are mean abundance values of the VOCs in samples acquired in duplicates with error bars as SEM. The star marks represents the *p*-value < 0.05. In panel B, the 3rd compound from left is 2,2,7,7-tetramethyltricyclo[6.2.1.0(1,6)]undec-4-en-3-one and the last compound is 1-4-hydroxy-3,5-di-*tert*-butylphenyl-2-methyl-3-morpholinopropan-1-one.

The validation cohort comprised external cohort of 32 IDC and 27 HC subjects. From the panel of 14 VOCs identified in the primary phase as statistically significant, five VOCs were validated in the external cohort of urine samples from IDC and HC subjects. The five VOCs were chosen based on the availability of the respective analytical standards in our laboratory. None of the down-regulated VOC standards could be obtained commercially and hence, the validation profiling was carried out for the five VOCs having an up-regulation pattern. The confirmation of the VOC panel was based on retention time and fragmentation pattern matching in the SIM mode. All the five VOCs showed the same pattern as observed in the primary phase experiments when analyzed on an independent cohort of patient samples. The expression pattern of the remaining nine VOCs that were identified as statistically significant differentially regulated in the initial cohort were found to maintain the same pattern in the external cohort examination. This was confirmed by the semi-quantitative chromatographic areas. The *p*-value significance for majority of the VOCs in the external cohort was found to be < 0.05 and is represented in Fig. 3 as star marks over the bars. Unfortunately, we also found some VOCs not following the significance criteria. The expression profile of the five SIM mode validated VOCs is represented as a bar graph in Fig. 3A and the semi-quantitative chromatographic area of the nine other VOCs is depicted in Fig. 3B.

### 3.4 Metabolic pathway analysis of the differentially expressed VOCs

**Fig. 4:**
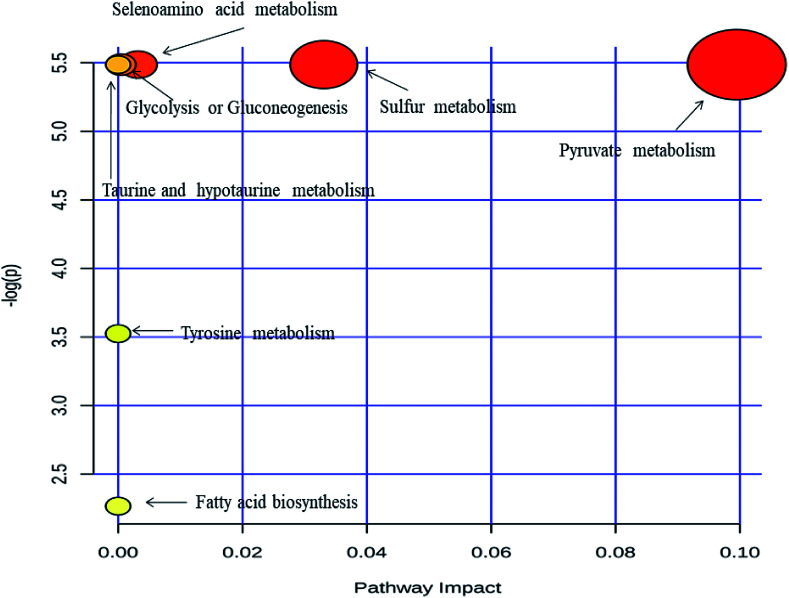
Metabolic pathway analysis performed by the MetPA tool in Metaboanalyst 3.0. Pathway topology analysis depicting dysregulated metabolic pathways in IDC patients.

Metabolic pathway analysis was carried out by MetPa tool of Metaboanalyst 3.0 web application. Most significantly altered pathways are enlisted in ESI Table S2† and showed in Fig. 4. The pathway analysis bubble plot (Fig. 4) comprises various matched pathways from the metabolome, which was arranged according to the *p*-values generated from pathway enrichment analysis. Similarly, the pathway impact values derived from the pathway topology analysis was calculated. These *p*-values were plotted on the *Y*-axis and the pathway impact values on the *X*-axis. The colour of the nodes corresponds to the *p*-values and the node radius is established through the pathway impact values. It is evident from the metabolic pathway analysis that all of the dysregulated pathways are excessively active in IDC as compared to the respective control subjects. Acetic acid emerged as prominent metabolite influencing majority of the dysregulated pathways. The role of acetate in malignant diseases is well established as an alternative energy source, epigenetic metabolite and a precursor to the fatty acid biosynthesis.^53^ Apart from acetic acid, dodecanoic acid is also upregulated in IDC subjects indicating enhanced lipid biosynthesis which is essential for malignant cell proliferation and tumor progression.^54^ Moreover, acetone, a well-known ketone body is detected at elevated concentration in urine of the breast cancer patients as compared to the control subjects. Ketone bodies are high energy fuel preferred by cancer cells under the hypoxic condition and therefore, it is not surprising to find acetone at a higher concentration in IDC patients.^55^

## 4. Discussion

This study was conceived with a rationale of identification of some volatile metabolites, which could be established as a panel of biosignature of IDC. Experimentally, we undertook a simple methodology to extract, analyze and identify volatile organic compounds in the urine samples of patients that showed statistically significant differential expression in IDC as compared to HC group. We employed HS-SPME extraction followed by GC-MS identification of urinary VOCs as a strategy to explore the urinary volatome and come up with statistically significant panel of VOCs that could segregate IDC subjects from HC. The statistical treatments applied to the urinary VOC data matrix revealed a panel of 14 VOCs (FC ≥ 1.5/≤ 0.67; *p*-value ≤ 0.05 and VIP score ≥ 1.0) which were responsible to discriminate IDC from HC group through the OPLS-DA model. Some of the potential candidate VOCs were further validated with analytical standards in a different cohort of patients revealing their consistent expression pattern suggesting them to be a useful panel for early diagnosis of IDC. It is necessary to understand that since most of the VOCs are secondary metabolites, the biochemical roles of these are less explored in humans. A few of the VOCs altered in IDC are interpreted for their associated biochemical roles according to the available literature, which are discussed henceforth.

2-Ethyl-1-hexanol is a member of the class of compounds known as fatty alcohols which are aliphatic alcohols consisting of a chain of at least six carbon atoms. Aldehydes or ketones are metabolized from hydrocarbons in the body with the help of activities of alcohol dehydrogenase (ADH) and cytochrome P450 enzymes.^56^ Phillips *et al.* related the elevation in the oxidative stress and enhanced activity of cytochrome P450 with BC pathology.^57^ The involvement of ADH, cytochrome P450 and oxidative stress in BC (in this case IDC) supports the fact that there is a conversion of hydrocarbons to alcohol. In this study, we have found 2-ethyl-1-hexanol to be more than six-fold upregulated with a VIP score of 1.2 in our analysis of urinary IDC samples and the same expression pattern was observed in the verification cohort. Câmara *et al.* recently showed elevated levels of 2-ethyl-1-hexanol in breast cancer cells using the SPME approach^34^ thereby strengthening the relationship of this VOC being indigenous to breast cancer. The HMDB suggests 2-ethyl-1-hexanol to be involved in membrane integrity and stability, energy storage, fatty acid and lipid metabolism pathways, and cell signaling.^58^ 1,2,3,4,5,6-Hexahydro-1,1,5,5-tetramethyl-2*s-cis*-2,4*a*-methanonaphthalen-7(4*aH*)-one, also commonly known as isolongifolenone was found to be more than 2 folds upregulated in IDC group with a VIP score of 1.75. Limited information is available for this VOC in terms of its biochemical relevance in cancer or other diseases. It has an inhibitory potential towards tyrosinase, which is a multifunctional copper-containing enzyme essential for melanin biosynthesis in animals and plants.^59^ Muthyala *et al.* reported the hydrogenated form of isolongifolenone as a critical ingredient for the preparation of chiral ligand for estrogen receptor, which could be expedient in prevention and treatment of breast cancer and other gynecological issues.^60^ 2-Methoxy-phenol, otherwise commonly known as guaiacol, is a methoxy group capped phenolic compound and is the monomethyl ether of catechol. According to the metabocard of guaiacol in the HMDB (HMDB0001398), it acts as an inducer of cell proliferation.^61^ It is found to be involved in the tyrosine metabolism and disulfiram pathway as per the HMDB.^58^ Guaiacol is reported to be found in the urine of patients with neuroblastoma and pheochromocytoma.^62^ Guaiacol was found to be overexpressed by 1.76 folds in IDC with respect to HC group and had a VIP score of 1.38 in our study. The up-regulation pattern of guaiacol in IDC is suggestive of its potential role in cell proliferation since it is one of the primary characteristics of the malignant cells. Further, HMDB suggests dodecanoic acid to be involved in biochemical pathways like fatty acid biosynthesis and beta-oxidation of very long chain fatty acid and also in cell signaling processes.^58^ Dodecanoic acid has been reported to be responsible for induction of apoptosis in colon cancer cells through oxidative stress induction.^63^ Lappano *et al.* recently reported the cellular signalling activated by dodecanoic acid in breast and endometrial cancer cells.^64^ Surprisingly, we found an up-regulation of 1.76 folds for dodecanoic acid, which had a VIP score of 1.08 in our study. Furan, a heterocyclic organic compound, is colorless, highly flammable and volatile liquid which has boiling point near the room temperature. It is not well studied in terms of its role in cancer and other disease pathologies. A study on murine models reported that higher doses of furan increase the chances of development of bile duct tumors in rats while a risk increment in hepatocellular tumors was observed in rat and mice.^65^ Furan was also found to be an important discriminator with a VIP score of 1.78 and was up-regulated by 1.60 folds in IDC group. Furan is well considered as a possible carcinogen^65^ and its detection in the urine samples of IDC patients strengthens the potential role in BC.

The above-discussed five VOCs were validated in an independent set of IDC urine samples. Apart from the referred VOCs, 9 other statistically significant differentially regulated VOCs are discussed henceforth for their potential role in cancer. 3-Methyl-phenol, is a methylated phenol useful in many chemical industrial applications. It has not been widely explored in terms of its relation to diseases. In the only study available, Ahmed *et al.* reported 3-methyl-phenol to be down-regulated in fecal samples of active Crohn's disease analyzed by similar SPME approach.^66^ Surprisingly, the data in our study revealed significant elevation in the expression of 3-methyl-phenol. It was found to be 1.89 times up-regulated in the urine samples of IDC patients as compared to healthy individuals. Similarly, another methyl derivative of phenol, namely 4-methyl-phenol also known as *p*-cresol was also found to be at elevated levels in the urine samples of IDC patients. It had a VIP score of 1.02 and a fold change ratio of 1.79. Sulphation and glucuronidation, part of conjugation process, are responsible for p-cresol being metabolized. Whereas, the elimination of the unconjugated *p*-cresol is partially through the urine. Hence, unsurprisingly *p*-cresol compound, along with various other phenols, gets retained in the kidney during kidney fail issues.^67,68^ It has been reported to affect various biochemical, biological and physiological functions such as diminishing oxygen uptake and blocking the cells K^+^ channels.^67^ Its excretion in elevated levels in IDC patients as compared to healthy controls suggests it could be potentially linked to IDC by some mechanisms, which needs to be explored further. Acetic acid was found to be 1.86 folds up-regulated with a VIP score of 1.30 in IDC group. It is often termed as one of the simplest carboxylic acids known. According to the HMDB, it is reported to be involved in different biochemical pathways like amino sugar metabolism, aspartate metabolism, fatty acid biosynthesis, pyruvate metabolism, ethanol degradation *etc.*^58^ Acetic acid has been reported to be associated with phenylketonuria, an inborn error of metabolism.^69^ Acetic acid was reported to be found in the urine samples of subjects in a study involving breast cancer and healthy controls.^40^ Filipiak *et al.* reported acetic acid to be found in the lung cancer tissue but did not find it in statistically significant levels.^36^ The enzyme ALDH has a function to oxidize acetaldehyde into acetic acid and reduced aldehyde levels are reported in few studies involving lung cancer.^70^ The increased level of acetic acid in our study suggests that there might be an elevated activity of ALDH in metabolizing acetaldehyde in IDC also. Phenol was observed as an important VOC, which was found to be up-regulated by 1.78 folds and had a VIP score > 1. It had a higher concentration in the urine samples of IDC patients when compared to that of healthy controls, thereby suggesting it might have been excreted in the urine samples of IDC upon being metabolized in the body. There is a report suggesting phenol to have tumor promotion properties on mouse skin.^71^ There is a factsheet which highlights that early life exposure to phenol and its other derivatives may lead to breast cancer risks in late years.^72^

Dimethyl trisulfide or DMTS is an organic compound and the simplest organic trisulfide. It was found to be 1.6 folds up-regulated in IDC group as compared to HC group with a VIP score of 1.05. It has been reported that in advanced cancer patients, there is a pungent sulfury malodour observed from the fungating cancer wounds and the researchers determined dimethyl trisulfide to be associated with it.^73^ The presence of DMTS in the urine samples of IDC can pave a way towards its potential possibility to be associated with IDC, which needs to be explored further. Ylangene, a sesquiterpenoid with three consecutive isoprene units and is classified under the lipid-like molecule class. In our study, we found this VOC to be down-regulated in the IDC urine samples with respect to HC urine samples. It had a fold change of 0.60 and 1.37 as VIP score. According to the HMDB, ylangene is involved in lipid peroxidation, lipid metabolism pathway and fatty acid metabolism.^58^ Ylangene-derived sesquiterpenoids from soft coral *Lemnalia philippinensis* has shown cytotoxic effects on HepG2, MDA-MB231 and A549 cancer cell lines.^74^ Therefore, the down-regulation of ylangene in IDC urine samples is quite justified as the subjects had a well-formed tumor. A few more furan derivatives were also found to be statistically significant differentially regulated but were related to food sources and hence have not been discussed in context to IDC. Some other VOCs were also found, the biochemistry of which is not documented anywhere and hence they are out of the scope of discussion. The expression profile of the statistically significant differentially expressed VOCs identified in the initial cohort didn't match exactly to the mathematical values of the ones reconfirmed in the external cohort. However, the expression pattern of the 14 VOCs in the external cohort was found to be matching with the initial cohort. This ascertains to the fact that the volatilome is very dynamic and is influenced by various confounders. Thus, identification of similar expression values needs a very closely controlled patient recruitment, which will eventually be helpful towards the establishment of VOCs as disease biosignature with confidence. As this study deals with VOCs, which are secondary metabolites, it is further needed to confirm the results obtained in this study in an even larger cohort of varied clinical samples to strengthen the potential of the biosignature for IDC type of breast cancer in a clinical scenario.

## 5. Conclusion

In summary, we undertook a simple methodology based on HS-SPME and GC-MS analysis to explore the urinary volatomic signature of IDC type of breast cancer. A urinary VOC signature of 14 compounds emerged as a statistically significant differentially regulated panel in IDC. The pathway analysis of this VOC panel revealed some biochemical pathways like pyruvate metabolism, glycolysis and gluconeogenesis, sulphur metabolism, taurine and hypotaurine metabolism, fatty acid biosynthesis, tyrosine metabolism, propanoate metabolism, synthesis and degradation of ketone bodies to be altered due to the alterations of the VOC biosignature. We further validated the expression pattern of five of these VOCs namely 2-ethyl-1-hexanol, isolongifolenone, furan, dodecanoic acid, 2-methoxy-phenol in another fresh cohort of urinary samples from IDC patients and found their expression pattern to be consistent with the primary sample set. Although a promising approach, this methodology needs to be explored further with a large cohort of patients to identify a key volatomic signature associated with IDC type of breast cancer, which could be effectively used in disease screening programmes in clinical setup across developing nations. When explored even further, molecular subtype-based disease biosignature of breast cancer could also be identified with this approach.

## Conflicts of interest

Authors declare no conflict of interest.

## Supplementary Material

RA-008-C8RA02083C-s001

RA-008-C8RA02083C-s002
