# Supplementary material for: A non-invasive approach to explore the discriminatory potential of the urinary volatilome of invasive ductal carcinoma of the breast
Source: RSC Adv. 2018 Jul 12;8(44):25040–50. doi: 10.1039/c8ra02083c (PMC9082450; doi:10.1039/c8ra02083c)

Electronic Supplementary Material (ESI) for RSC Advances.
This journal is © The Royal Society of Chemistry 2018

Supplementary Table S1: The clinical and demographic information of the IDC subjects and healthy controls.

**Characteristics**	**IDC (Discovery cohort / External cohort)**	**Healthy control (Discovery cohort / External cohort)**
Biological Specimen	Urine	Urine
Number of participants (male / female)	65 (0,65) / 32 (0, 32)	70 (0,70) / 27 (0,27)
Age, median (range)	54 ± 8 (27-80) /	50 ± 10 (25-74) /
Eastern India residence	12 / 7	6 / 6
Western India residence	32 / 12	25 / 9
Northern India residence	15 / 5	21 / 8
Southern India residence	6 / 8	18 / 4
BC type Luminal A	19 / 7	N.A. / N.A.
BC type Luminal B	28 / 12	N.A. N.A.
BC type HER2 Enriched	13 / 11	N.A. / N.A.
BC type Basal	5 / 2	N.A. / N.A.
Diet (Veg/Non-Veg)	(27 / 38) / (18 / 14)	(36 / 34) / (17 / 10)

Supplementary Table S2. The most significantly altered pathways observed in IDC which evolved from the MetPA analysis.

Sr. No.	Pathway name	Hits (FC)	p-value	FDR
1	Pyruvate metabolism	Acetic acid (0.896918)	0.006421	0.011558
2	Sulfur metabolism	Acetic acid (0.896918)	0.006421	0.011558
3	Selenoamino acid metabolism	Acetic acid (0.896918)	0.006421	0.011558
4	Glycolysis or Gluconeogenesis	Acetic acid (0.896918)	0.006421	0.011558
5	Taurine and hypotaurine metabolism	Acetic acid (0.896918)	0.006421	0.011558
6	Tyrosine metabolism	Phenol (0.830554)	0.042958	0.064437
7	Fatty acid biosynthesis	Dodecanoic acid (0.668212), n-Decanoic acid (NA)	0.1037	0.1126

Supplementary Table S3. The list of all the VOCs identified in IDC subjects and healthy controls.

Supplementary Fig. S1. Data normalization in Metaboanalyst 3.0 where the dataset was quantile normalized, cube root transformed and range scaled after missing value imputation via Bayesian PCA algorithm.

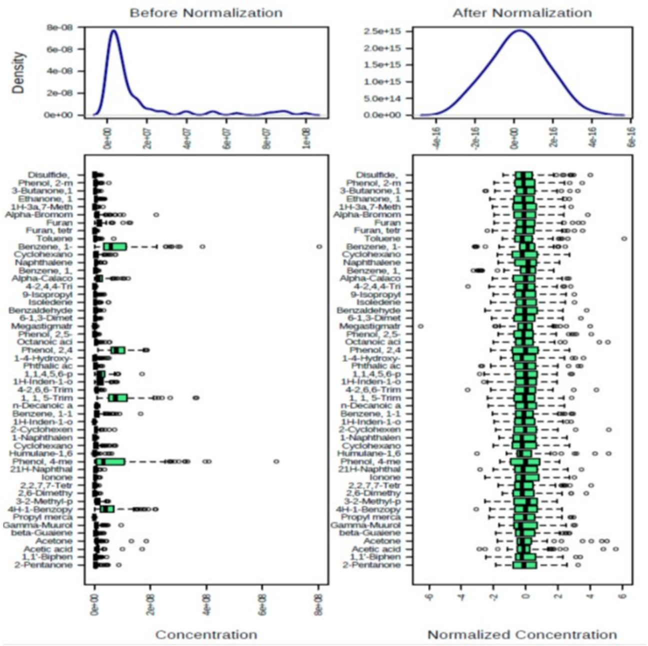

Supplement: RA-008-C8RA02083C-s001 [file RA-008-C8RA02083C-s001.pdf]
